# SCAR Marker for Gender Identification in Date Palm (*Phoenix dactylifera* L.) at the Seedling Stage

**DOI:** 10.1155/2018/3035406

**Published:** 2018-10-17

**Authors:** Fahad Al-Qurainy, Abdulhafed A. Al-Ameri, Salim Khan, Mohammad Nadeem, Abdel-Rhman Z. Gaafar, Mohamed Tarroum

**Affiliations:** ^1^Department of Botany and Microbiology, College of Science, King Saud University, Riyadh 11451, Saudi Arabia; ^2^Department of Biology, Faculty of Education and Science, Rada'a Al-Baydha University, Al-Baydha, Yemen

## Abstract

Date palm (*Phoenix dactylifera* L.) is cultivated in arid and semiarid regions worldwide. Given the dioecious nature of this plant, gender identification is very important at the seedling stage. Molecular markers are very effective tools that help in gender identification at this stage. A sequence characterized amplified region (SCAR) marker linked to sex-specific regions in the genome of date palm was developed. Of the 300 tested randomly amplified polymorphic DNA (RAPD) primers, only one primer (OPC-06) produced reproducible band (294 bp) in male plants. The PCR product of this primer was cloned and sequenced. The specific primers were synthesized for amplification of a 186 bp fragment in male date palm plants. These primers were validated in male and female date palm plants, wherein the designed SCAR marker was reported only in male plants and no amplification was observed in female plants. The developed SCAR marker was used with seedlings of date palm and proved very effective in identification of gender.

## 1. Introduction

Date palm (*Phoenix dactylifera* L.) belongs to the family Arecaceae (2n = 36) and has a socioeconomic significance. The plant is cultivated for food, fiber, and shelter in different arid and semiarid regions worldwide. It is a monocot and dioecious tree (separate male and female) and serves as an important commercial crop in Middle Eastern countries. The plant is native to the Canary Islands located in the Atlantic Ocean near the coast of Northeast Africa. Dates are a good source of energy, vitamins, and group of elements such as phosphorus, potassium, iron, manganese, selenium, zinc, and calcium [1, 2].

Research related to date palm is greatly restricted, owing to the lack of measures to identify its gender at the seedling stage. Date palm cultivation is more cost-effective through the cultivation of female plants than male plants. An increase in the number of female date palm plants per hectare may result in an increase in date production, thereby making the plantation more profitable. This has prompted the farmers to solely propagate date palm cultivars via offshoots that results in the reduction in genetic variations. The high genetic diversity is very important in plants for their survival in their natural habitat [3]. However, the seedlings produced may be either male or female, and no reproducible technique is currently available for gender determination in germinated seeds of date palm. Several efforts have been directed recently to establish a method for the early detection of seedling gender before their plantation in fields. However, no methodology has so far been developed for gender identification at the seedling stage [2].

Molecular markers based on the direct analysis of genomic DNA are used for the study of phylogenetic relationship, genetic diversity, genetic fidelity, and genotoxicity of date palm cultivars [4–7]. These markers may be useful in the study of sex determination in dioecious plants. Despite increasing research efforts on a number of different plant species, very limited information is available on the molecular basis of gender determination, and it may be very difficult to estimate the numbers of genes involved. However, in some plant species, sex-determining genes have been discovered including *Carica papaya* and *Asparagus officinalis* [8, 9]. In the last two decades, efforts have been made to understand the basis of gender determination in date palm and develop methods for gender identification at an early stage using isozymes [10], peroxidases [11] and DNA-based molecular markers using random amplified polymorphic DNA (RAPD) [12], and polymerase chain reaction-based restriction fragment length polymorphism (PCR-RFLP) [13]. The first genetic map of Khalas cultivar of date palm has been published [14] which could help in understanding the sex chromosome development. The sex chromosomes evolved from a common autosomal origin before the diversification of the extant dioecious *Phoenix* species [15]. DNA-based marker linked to sex determination locus in *Salix viminalis* was used for estimation of sex ratios in progeny [16]. In comparison with other molecular markers, RAPD markers offer advantages owing to their ease of generation and suitability for genetic polymorphism study in different plant species that lack detailed genomic sequence information [17]. However, there are some limitations with the use of RAPD markers as PCR amplification is very sensitive and depends on many factors. For more reproducible results, RAPDs may be converted into stable and reliable markers through the cloning of amplified bands, sequencing, and designing of more specific primers. Annealing of these specific primers under stringent annealing temperatures in PCR may result in the production of a single band that corresponds to genetically defined loci, sequence-characterized amplified regions (SCAR) [18]. This approach has been employed to develop several gender-linked molecular markers in dioecious plants, including *Silene latifolia* [19], *Pistacia vera* [20], *Cannabis sativa* [21], *Humulus lupulus* [22], *Actinidia chinensis* [23], *Atriplex garrettii* [24], *Carica papaya* [25], *Salix viminalis* [26], *Rumex acetosa* [27], *Mercurialis annua* [28], and *Eucommia ulmoides* [29].

Here, we developed a SCAR marker specific to male date palm plant. The male specific bands were generated through the comparative study of male and female plants using RAPD primers. The designed primers specific to male plants (SCAR primers) were used for the identification of gender at the seedling stage in date palm.

## 2. Materials and Methods

### 2.1. Plant Material Collection

Leaf samples of date palm (21 different males and females) were collected from Al-Rajhi Farm (Al-Qassim) and Agricultural Research Station (Dirab) in Saudi Arabia and stored at −80°C (Table 1).

### 2.2. Genomic DNA Extraction

Genomic DNA was isolated from the leaves using the modified CTAB method [30]. The leaves of date palm (200 mg) were ground into fine powder with a mortar using liquid nitrogen. The frozen powdered tissues were transferred into a 2 mL microcentrifuge tube and treated with 800 *μ*L of preheated extraction buffer and 10 *μ*L of RNase A (10 mg/mL) (Qiagen). To the above mixture, 100 *μ*L of 3% PVP and *β*-mercaptoethanol was added and followed by its incubation at 65°C for 20 min with shaking every after 5 min. The mixture was then cooled at room temperature and treated with an equal volume of chloroform and isoamyl alcohol (24 : 1), followed by its frequent mixing for 20 min. The mixture was subjected to centrifugation at 10,000 rpm for 10 min at room temperature. The clear upper aqueous suspension was transferred into a new microfuge tube and treated with an equal volume of ice-cold isopropanol at −20°C for 1 h. For the separation of nucleic acid, tubes were centrifuged at 10,000 rpm for 10 min. The supernatant was discarded, and the pellet was washed twice with cold 70% ethanol. The DNA pellet was dried at 37°C and dissolved in 200 *μ*L of TE buffer (Qiagen).

### 2.3. RAPD Analysis

We performed PCR reaction with genomic DNA of male and female date palm plants to screen 300 decamer primers of arbitrary sequences (Operon Technologies, United States). For each primer, genomic DNA from two pools (21 female cultivars in one pool and 21 male plants in second pool) were used for PCR. PCR amplifications were performed in 20 *μ*L reaction volumes containing 5x HOT FIREPol® Blend Master Mix ready to load (4 *μ*L), primer (15 ng/*μ*L), template DNA (25 ng/*μ*L), and water. DNA amplification was performed on Applied Biosystems Veriti 96-well Thermal Cycler with the following program: first denaturation at 94°C for 5 min, followed by 40 cycles of denaturation for 1 min at 94°C, annealing at 36°C for 1 min, extension at 72°C for 1 min, and a final extension step at 72°C for 5 min. Amplification products were analyzed by gel electrophoresis on 1.3% agarose gel in 1x TBE buffer (*Tris/borate/ethylenediaminetetraacetic acid*). Gels were stained with ethidium bromide and visualized under UV light. Each amplification reaction was performed using a single primer and repeated thrice to verify the reproducibility of the results.

### 2.4. Identification of Male Specific Band from RAPD Profile

A total of 300 decamer oligonucleotides (RAPD primers) were used with the bulk DNA of male and female plants for selection of male specific band. The male specific primer obtained in screening with bulk DNA samples was used further with individual DNA sample of male and female plants for reproducibility testing and selection of male specific band.

### 2.5. Cloning and Sequencing of Male Specific Band Generated in RAPD Profile

The candidate RAPD fragment specific to male plant was carefully excised from 1.2% agarose gel using a sterile gel slicer and purified with kit Wizard® SV Gel and PCR clean-up system (Promega). The male specific band was cloned and sequenced using the commercial service offered by *Macrogen* Inc. (Korea). The sequence was subjected to BLAST at NCBI database to determine its similarity with the available sequences (https://www.ncbi.nlm.nih.gov/).

### 2.6. Designing of SCAR Primers

The ends of the cloned RAPD fragment were used to design specific primers (SCAR primers) for the amplification of the expected size from the genomic DNA of male date palm plants. However, these specific primers were designed from the male-specific sequence using primer 3 tool (http://frodo.wi.mit.edu/) as well as Primer Select software (DNASTAR).

### 2.7. Validation of Developed SCAR Marker

Amplification of the genomic DNA from male and female plants was performed with SCAR primers in a 25 *μ*L reaction volume using the master mixture. Designed primers were used for the amplification of SCAR marker using the genomic DNA of known cultivars (Table 1). The single reaction mixture contained Illustra PuReTaq Ready-To-Go PCR Beads (4 *μ*L), 20 ng of each primer (forward and reverse), 25 ng of template DNA, and water. The PCR program followed as first denaturation at 94°C for 4 min, followed by 40 cycles of denaturation at 94°C for 1 min, annealing at 55°C for 30 s, extension at 72°C for 1 min, and final extension at 72°C for 5 min. Amplification products were separated on 1.5% agarose gel.

### 2.8. Screening of Gender in Seedlings of Date Palm

The designed SCAR marker was employed for the identification of date palm gender in two-month-old seedlings. The seedlings of Khalas cultivar were screened for gender identification using designed SCAR marker.

## 3. Results and Discussion

### 3.1. RAPD Analysis

**Figure 1:**
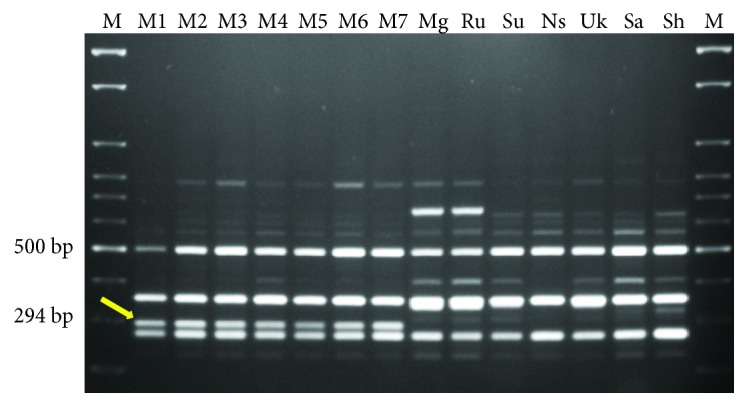
Amplification profile generated with RAPD primer (OPC-06) using genomic DNA of male and female date palm plants. Lane M, 100 bp ladder.

**Figure 2:**
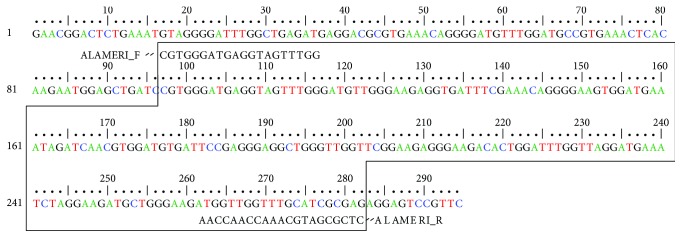
Complete DNA sequence of the cloned RAPD fragment specific to male plant of *Phoenix dactylifera*. ALAMERIF and ALAMERIR are specific forward and reverse SCAR primers.

In the preliminary study, 300 arbitrary decamer RAPD primers were screened using bulk DNA from 21 female cultivars (one pool) and 21 male plants (second pool) (Table 1). Of these primers, only OPC-06 (5′-GAACGGACTC-3′) produced a band of approximately 294 bp specific to male plants. After confirmation of male specific band in bulk samples, further PCR reaction was performed using DNA of individual female and male plant as result shown in Figure 1. The presence of 294 bp band was clearly observed in all male plants but absent in female plants. This male-specific fragment amplified by the primer OPC-06 was subsequently excised, purified, cloned, and sequenced. Homology search was performed using BLAST algorithm of 294 bp sequence (Figure 2), and no similarity was found with any of the known sequences from NCBI GenBank database.

### 3.2. Development and Validation of SCAR Marker

**Figure 3:**
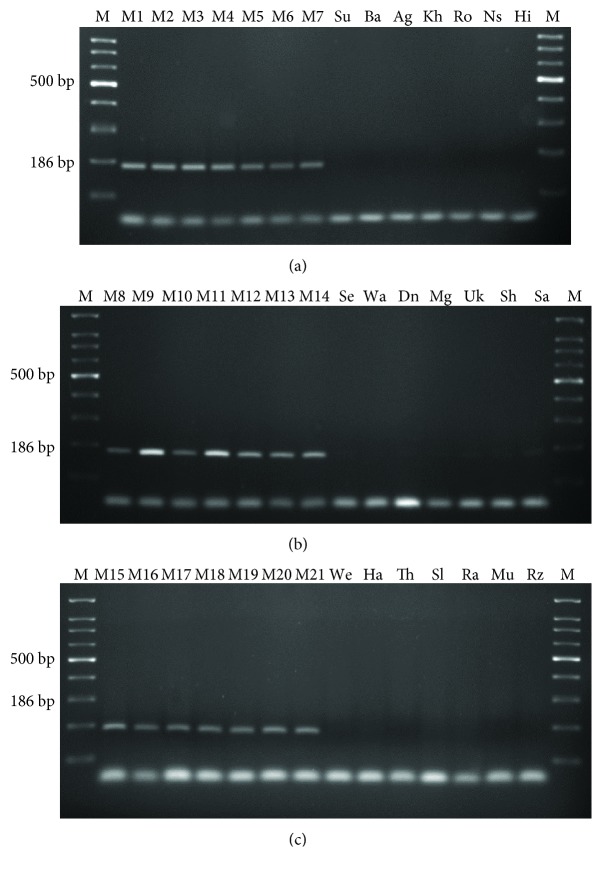
SCAR marker analysis using SCAR primers showed the amplification of a 186 bp fragment in all male plants and absent in female plants. Lane M, 100 bp ladder. (a) M1–M7 (male), Su-Hi (female). (b) M8–M14 (male), Se-Sa (female). (c) M15–M21 (male), We-Rz (female).

A pair of SCAR primer was designed from male-specific sequence obtained in RAPD profile, to amplify a 186 bp fragment (Figure 2). PCR reaction was performed with genomic DNA of individual male and female plants with designed SCAR primer pair “ALAMERI” (ALAMERIF 5′-CGTGGGATGAGGTAGTTTGG-3′ and ALAMERIR 5′-CTCGCGATGCAAACCAACCAA-3′). A single, distinct bright band of size 186 bp was observed in all male plants whereas was absent in all female plants (Figures 3(a)–3(c)). Thus, RAPD marker was successfully converted into SCAR marker. The reproducibility of the developed SCAR marker was verified on 55 samples with known gender other than the previously tested male and female plants. The marker clearly differentiated all male from female plants based on the presence or absence of the 186 bp band. The results were reproducible owing to the longer length (20-21 base) and high Tm (60.5–61.3°C) of primers. Thus, the developed SCAR marker showed reproducible results, as specific band was obtained only in male plants.

The SCAR marker has been used for gender identification in many dioecious plant species in which male and female plants look similar at vegetative stage. Male-specific SCAR markers were developed in dioecious plants such as *Humulus scandens*, *Rumex nivalis*, and *Phoenix dactylifera* using molecular marker profiling of male and female plants [31–34]. The seeds of *Pistacia chinensis* produce biofuel. To enhance the number of female plants of *P. chinensis* for more fuel production, SCAR marker specific to female plants was developed for identification at seedling stage [35]. SCAR markers have been used to discriminate between male and female plants of *Hippophae rhamnoides* [36].

**Figure 4:**
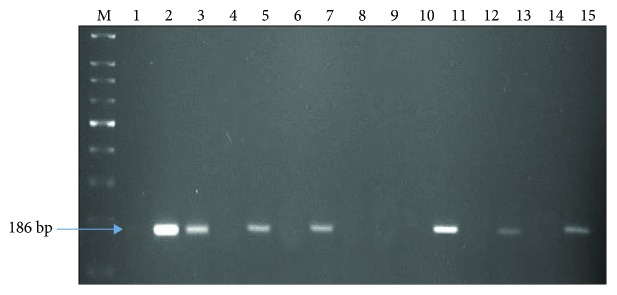
Screening of male and female plants in date palm seedlings with developed SCAR marker. Presence of band indicates male seedlings and absence of band indicates female seedlings.

At seedling stage, all date palm plants appear similar in morphology and it is very difficult to identify as male or female among them. The presence or absence of the SCAR marker developed herein could allow differentiation between male and female plants at the seedling stage [37]. We screened two-month-old Khalas seedlings with our developed SCAR marker. The SCAR marker was present in all male plants but absent in female plants (Figure 4). The lanes 2, 3, 5, 7, 11, 13, and 15 corresponded to male seedlings, whereas lanes 1, 4, 6, 8, 9, 10, 12, and 14 had all female seedlings (Figure 4).

In conclusion, the developed SCAR marker could be used for gender identification at the seedling stage of date palms to save time, as the plant takes 5–7 years to reach its reproductive stage. Thus, plant breeders may adopt this marker as a potential tool for gender identification of date palm seedlings before their plantations in fields.

## Figures and Tables

**Table 1:** Date palm leaves collected from female cultivars and male plants.

S.N.	Cultivar (female)	Cultivar code	Male	Male code
1	Barhi	Ba	Male-1	M1
2	Seqae	Se	Male-2	M2
3	Sukkari	Su	Male-3	M3
4	Sabaka	Sa	Male-4	M4
5	Wannana	Wa	Male-5	M5
6	Khalas	Kh	Male-6	M6
7	Ruthana	Ru	Male-7	M7
8	Deglet Noor	Dn	Male-8	M8
9	Magdool	Mg	Male-9	M9
10	Agwa	Ag	Male-10	M10
11	Um Khashab	Uk	Male-11	M11
12	Hilaly	Hi	Male-12	M12
13	Shaishee	Sh	Male-13	M13
14	Naboot Seif	Ns	Male-14	M14
15	Ruzeiz	Rz	Male-15	M15
16	Wesaily	We	Male-16	M16
17	Sullaj	Sl	Male-17	M17
18	Thawee	Th	Male-18	M18
19	Hatmi	Ha	Male-19	M19
20	Rabeaa	Ra	Male-20	M20
21	Munif	Mu	Male-21	M21

## Data Availability

The quantitative data and graphical (pictures) used to support the findings of this study are included within the article.
